# Insulation and Evaporative Resistance of Clothing for Sugarcane Harvesters and Chemical Sprayers, and Their Application in PHS Model-Based Exposure Predictions

**DOI:** 10.3390/ijerph17093074

**Published:** 2020-04-28

**Authors:** Kalev Kuklane, Róbert Toma, Rebekah A.I. Lucas

**Affiliations:** 1 Thermal Environment Laboratory, Division of Ergonomics and Aerosol Technology, Department of Design Sciences, Lund University, Box 118, SE-22100 Lund, Sweden; 2 Institute for Safety (IFV), P.O. Box 7112, 2701 AC Zoetermeer, The Netherlands; 3 Energy Institute, Brno University of Technology, 601 90 Brno, Czech Republic; 145743@vutbr.cz; 4 School of Sport, Exercise and Rehabilitation Sciences, University of Birmingham, Birmingham B15 2TT, UK; R.A.I.Lucas@bham.ac.uk; 5 La Isla Network, Washington, P.O. Box 816, District of Columbia, MI 49301, USA

**Keywords:** heat stress, dehydration, protective clothing, sugarcane field workers, prevention, clothing insulation, evaporative resistance, predicted heat strain, exposure evaluation, human thermal modeling

## Abstract

Many workers are exposed to heat stress that can be exacerbated by the type of clothing they wear. The resulted heat strain can lead to short or long-term heat-related disorders. This study aimed to measure clothing properties of sugarcane field workers and evaluate the heat strain by an international standard, predicted heat strain model (PHS). The clothing thermal insulation and evaporative resistance values of sugarcane cutter and chemical sprayer outfits were acquired for the whole body, body regions and specific body parts via thermal manikin measurements. The detailed clothing insulation values of body parts can be utilized in advanced thermo-physiological models, while in this study, the values for the whole body together with weather data were used in PHS. Estimated duration limited exposure times (DLE) for an hour-by-hour prediction over a workday and for a range of high humidity scenarios were calculated. Such evaluation tools can be used for risk assessment and management to support organizational measures and prepare equipment and materials in the case of hot weather events in order to avoid dehydration and other heat-related disorders.

## 1. Introduction

Agricultural jobs are very much dictated by season and climate. This type of work needs to be done at specific times of the year and often these jobs are connected with warm or warm and wet seasons. Jobs related to sugarcane production are no exception. Sugarcane production in some parts of the world still requires heavy manual labor in a hot environment. Repeated heat exposure together with insufficient water replacement has been related to chronic kidney disease of unknown etiology (CKDu) [1,2]. CKDu is associated with a high mortality rate and has reached epidemic levels in several tropical countries, including Latin America, e.g., Nicaragua, El Salvador, etc. According to Moran and Gaffin [3] with reference to Knochel [4] and Knochel and Reed [5], heatstroke results in a 25% higher risk of kidney failure. If heatstroke does indeed increase the risk of kidney failure, then long-term exposure that is close to heat tolerance limits may do so as well. Industrial sugarcane workers perform difficult, strenuous work under hot environmental conditions. All measures to reduce heat stress and improve the situation for these agricultural workers are needed. It is possible that clothing and protective gear further exacerbate worker’s heat exposure, as clothing has a strong thermal impact on humans. The insulation and evaporative resistance can have opposing effects on thermal balance: the insulation effect can limit the radiant heat load, while the evaporative resistance impairs sweat evaporation and thereby promotes heat storage. Additionally, body motion creating a pumping effect in clothing at approximately similar workloads may allow lower thermal stress than in more static tasks due to enhanced evaporation. In connection to the changing climate, the human thermo-physiological and clothing models would allow making long-term impact predictions on humans based on climate change models. In order to reduce protective clothing-induced stress, we need to know the thermal performance of the available clothing items and other protective gear. The objectives of this study were to:

(a) measure the insulation and evaporative resistance of the clothing used in the agricultural sector for the sugar industry for two tasks—sugarcane harvesting and chemical spraying and-
(b) utilize the outcome in a predicted heat strain model (PHS) according to an international standard [6] for allowed exposure time prediction and recommendations.

## 2. Materials and Methods

In order to meet the objectives, the study was split into two sections. The first part dealt with measurements of clothing insulation and evaporative resistance. The second part utilized the acquired clothing properties and available information on the working conditions to predict the heat stress in selected conditions that may allow for preventive-measures planning.

### 2.1. Measurements of Clothing Properties

**Figure 1:**
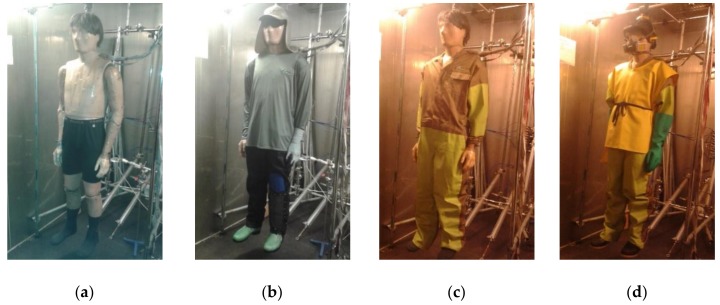
Tested clothing: (**a**) underwear for both systems (provided by the laboratory), (**b**) sugarcane harvester’s outfit (glove only on one hand and leg protector on one leg), (**c**) chemical sprayer’s protective coveralls on top of underwear and (**d**) chemical sprayer’s complete outfit with outer protective layers.

The thermal manikin Tore at Lund University, Sweden [7,8] was used for testing. Tested clothing ensembles were acquired from Ingenio San Antonio, the largest sugar mill in Nicaragua, and are currently worn by field workers (Figure 1). The sugarcane cutter (SC) outfit consisted of boxer shorts, socks, jeans, synthetic long-armed shirt, cap with textile for neck protection, protective boots, eye protection of metal mesh, glove on one hand and leg protection made of metal grid on one leg, with a total weight of 2.8 kg. The chemical (pesticide) sprayer (CP) outfit consisted of boxer shorts, socks, partially impermeable coveralls, impermeable apron covering front and back, protective gloves, cap, respirator and protective boots, with a total weight of 4.1 kg.

#### 2.1.1. Clothing Insulation

The clothing insulation was measured following the methods described in standards ISO 15831 [9] and ISO 9920 [10] with some modifications. The tests were carried out at 20.0 ± 0.1 °C with 0.21 ± 0.08 m/s air velocity in static (*I*_T_) and dynamic (*I*_T,r_, walking 90 steps/min, corresponding to the speed of about 3.5 km/h) conditions. Clothing basic insulation (*I*_cl_) for PHS predictions was calculated according to the standards, considering air layer insulation (*I*_a_) and clothing area factor (*f*_cl_). The latter was acquired from a photographic method taking pictures of the garment ensembles and the nude manikin from front and from the side [11]. For basic information for evaporative resistance calculations, the insulation of the textile, simulated sweaty skin was also measured but only in static conditions. Air layer insulation around the human body shape was measured with a nude manikin. Insulation was measured with hair (wig) on the manikin’s head.

#### 2.1.2. Evaporative Resistance

Total evaporative resistance (*R*_et_) of the clothing sets and the wet textile skin was measured following ASTM F2370-15 [12] at so-called isothermal conditions with the manikin surface and air temperature being set to 34 °C and utilizing the heat-loss method for evaporative resistance calculations. Evaporative resistances were measured without using a wig on the manikin’s head, and the air velocity was kept at 0.54 ± 0.09 m/s in order to avoid build-up of water vapor pressure in the air near the manikin surface that could affect the evaporation. The evaporative resistance values were corrected for differences in the manikin surface and textile skin based on heat loss according to Wang et al. [13].

#### 2.1.3. Data Presentation

Each condition was tested twice. In order to utilize the insulation and evaporative resistance values in advanced thermal models, insulation or evaporative resistance of individual body areas are needed, as thermoregulatory responses (i.e., blood flow and sweating) are heterogeneous across the body and not uniformed. Therefore, an average value for a clothing set does not allow proper evaluation of localized discomfort, though simple and low-cost models, including the PHS, utilize an average whole-body value for a complete clothing set. Therefore, the results are presented as average values for individual zones, regional areas and an average whole-body value.

The insulation results are sorted by the percentage differences of air layer insulation in static as compared to dynamic conditions. The evaporative resistance results are sorted by the magnitude of difference between measured and corrected wet textile skin total evaporative resistance values.

### 2.2. Exposure Evaluation According to Predicted Heat Strain Model (PHS)

**Figure 2:**
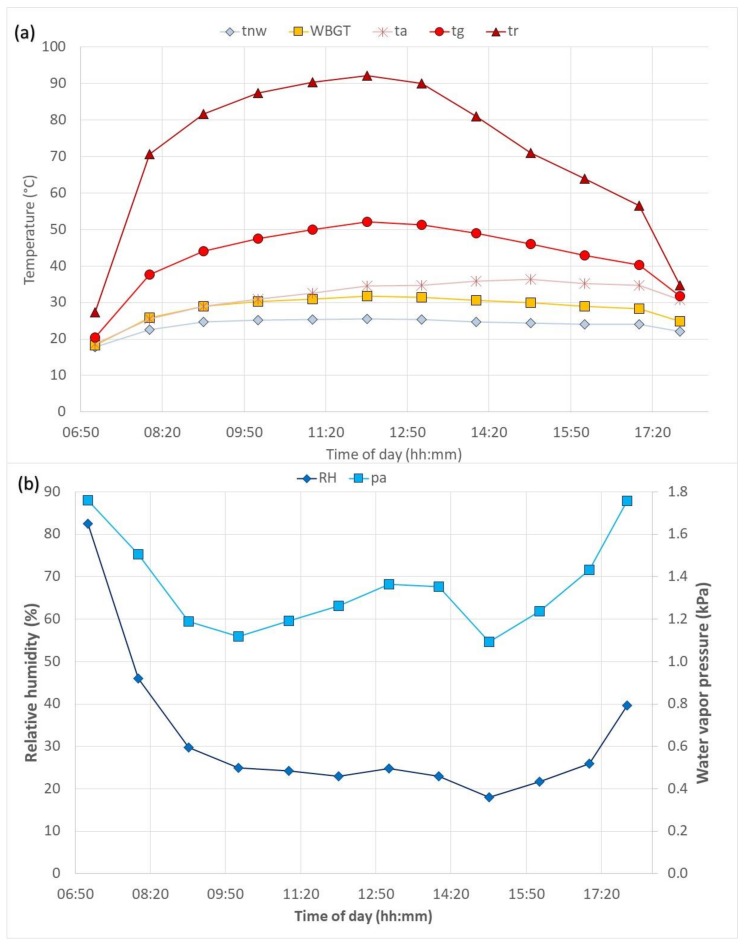
Environmental parameters for one day (14 January 2015) in a sugarcane field in Nicaragua. (**a**) Temperatures (°C): *t*_nw_—natural wet bulb temperature, WBGT—wet bulb globe temperature index; *t*_a_—air temperature, *t*_g_—temperature of a globe thermometer and *t*_r_—mean radiant temperature; (**b**) humidity: RH—relative humidity (%) and *p*_a_—absolute humidity expressed as water vapor pressure in the air (kPa). Average wind speed over the day was relatively stable around 2 m/s.

In spite of criticism on the PHS model [14,15,16], we considered that the PHS model is easily available for everybody, has a low cost and has been validated in a wide range of hot conditions. If to consider the limitations related to heavily insulating clothing [16] and repeated exposures [14,15], it still gives a reasonably good prediction, that is very useful for planning a workday in advance and preparing preventive measures against heat stress. Thus, a web tool based on PHS algorithms [6] at http://www.eat.lth.se/fileadmin/eat/Termisk_miljoe/PHS/PHS.html was utilized for the exposure evaluations. The programmer had access to ISO/FDIS 7933: 2004; however, it was verified that the DIS version and final standard [6] were the same. Exposure characteristics were calculated as the limit values based on the core temperature and water loss based on an hour-by-hour approach for one hot day weather conditions (Ta = 18.6–36.4 °C, *T*_g_ = 20.5–52.1 °C; see Figure 2) for several activity level combinations. Predictions were made for each hour conditions and did not reflect the physiological status of the previous hour. Thus, the predictions for the morning period may overestimate the duration limited exposure (DLE), while afternoon periods may underestimate it.

Additionally, a range of temperature conditions (Ta = 28–36 °C) was selected as a fictive pedagogical example where each selected workload, in combination with the tested clothing, would show some DLE for the core temperature. Thus, in this simulation, the selected temperatures were combined with the same high constant relative humidity (70%). The whole range of set conditions would not be relevant for specific agricultural activities. Instead, some of these may be relevant for specific process industries; for example, the paper industry, where some urgent tasks have to be carried out at high temperatures and humidity, or the glass industry, where similar clothing (except for specific leg and hand protections) may be used. The selected conditions with high temperatures and humidity may also resemble the conditions in restaurant kitchens and laundries in warm countries [17].

From manikin tests, the clothing properties (basic insulation and evaporative resistance; for methods, see Section 2.1) for both sets were were close to the limits of PHS requirements (*I*_cl,SC_ = 0.107 m^2^ K/W, *I*_cl,CP_ = 0.177 m^2^ K/W; *R*_ecl,SC_ = 13.2 m^2^ Pa/W, *R*_ecl,CP_ = 74 m^2^ Pa/W). Clothing properties and hourly sugarcane field weather data were used in the PHS tool to evaluate the heat stress in the present conditions for one day (Figure 2). For the high humidity scenario, the wind speed (*v*_a_) was kept 2 m/s as measured in the field today, while relative humidity (*RH*) was set to 70%, allowing water vapor pressure in the air (*p*_a_) to change depending on air temperature. Additionally, the mean radiant temperature (*t*_r_) 70 °C and alt. globe temperature (*t*_g_) 44 °C were kept constant for this scenario. Estimated activity levels (200–300 W for CP and 250–350 W for SC) and other required parameters were also entered into the PHS model. Activity levels were based on heart rate measures taken from sugarcane workers in the field and estimated according to ISO 8996 [18], as well as from previous measurements and estimations in the field [17]. The workers were assumed to be acclimated, and drink was freely available. For predictions, some anthropometric parameters were taken as estimated averages. It was also assumed that workers were frequently in a standing posture with an average walking velocity (*v*_w_) of 0.5 m/s. Table 1 lists most of the input parameters; however, some parameters are not listed, as they are mentioned in the text above or did change depending on other input data; for example, water vapor pressure in the air was calculated by air humidity and air temperature.

## 3. Results

### 3.1. Manikin Tests

Clothing area factors from the photographic method for SC and CP were 1.26 and 1.41, respectively. Clothing total and total resultant insulation for different body parts, body regions and for the whole ensembles (marked as “total”) values for the air layer, sweating skin and tested ensembles are given in Table 2. Clothing total evaporative resistance for body parts, body regions and for the whole ensembles are given in Table 3.

### 3.2. Evaluation of Heat Strain with PHS

**Figure 3:**
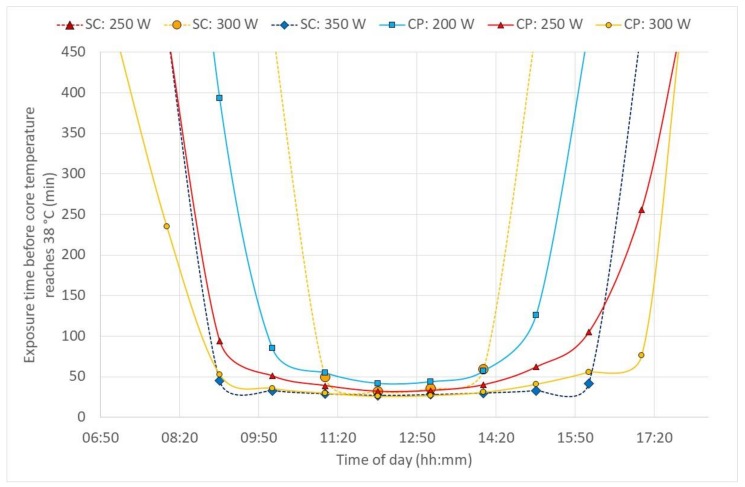
Expected daily duration limited exposure (DLE) for sugarcane cutters (SC) and chemical sprayers (CP) at various activity levels based on core temperature (criterion *T*_rec_ < 38 °C). At the lowest activity for sugarcane cutters (SC: 250 W), DLE was above 8 h (480 min), and therefore, the line cannot be seen in this diagram.

**Figure 4:**
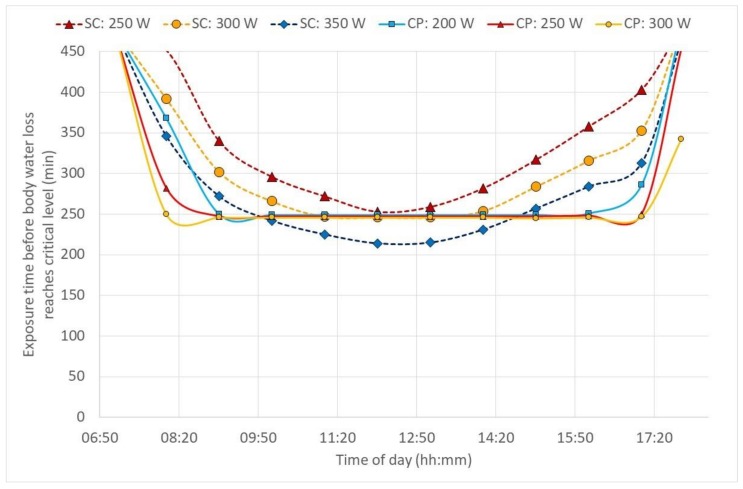
Expected daily duration limited exposure for sugarcane cutters (SC) and chemical sprayers (CP) at various activity levels based on water loss (criterion *D*_wl,lim_ < 5%).

Figure 3 and Figure 4 show the duration limited exposure (DLE) based on core temperature and water loss criteria, respectively, during one day hour-by-hour in sugarcane cutter and chemical sprayer outfits. Although, outdoor work has a changing environment and PHS cannot really accommodate that, still the example here, reflected in Figure 3 and Figure 4, is an attempt to show a possibility to manage that shortcoming with hour-by-hour calculations based on the environmental data given in Figure 2.

**Figure 5:**
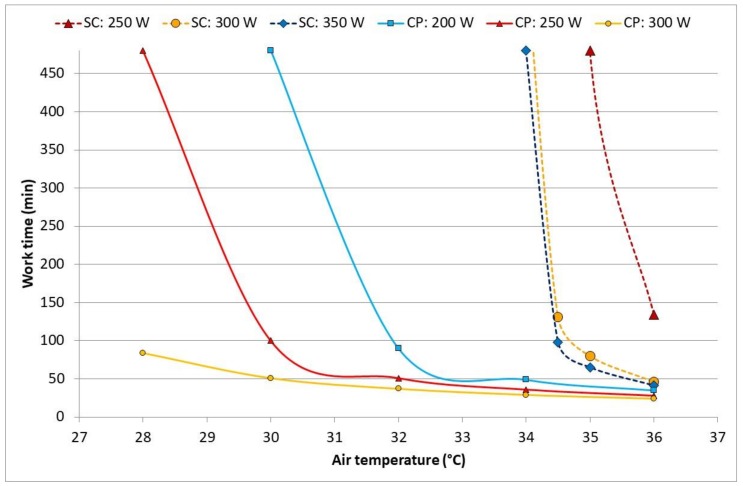
Expected duration limited exposure for sugarcane cutters (SC) and chemical sprayers (CP) at various activity levels based on core temperature (criterion *T*_rec_ > 38 °C) in a fictive high humidity scenario.

**Figure 6:**
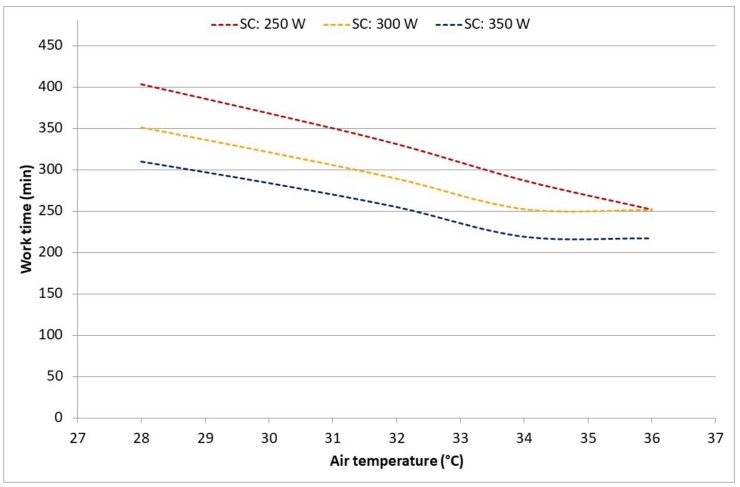
Expected duration limited exposure for sugarcane cutters at various activity levels based on water loss (criterion *D*_wl,lim_ < 5%) in a fictive high humidity scenario.

Figure 5 shows the DLE based on the core temperature depending on the air temperature (air humidity was always set to 70% independent of the temperature) and workload for SC and CP, respectively. Figure 6 shows the DLE based on water loss for SC. For CP, the core temperature was always the major limiting parameter, and the water loss limit stayed commonly the same for almost all conditions around 250 min. Here, it has to be pointed out that, for a practical application, the lowest DLE value has to be selected as an allowed work limit.

## 4. Discussion

### 4.1. Manikin Tests

Total and total resultant clothing insulation for the air layer (AL), sugarcane cutters (SC) and chemical sprayers (CP) were 0.098 and 0.076, 0.191 and 0.143 and 0.257 and 0.188 m^2^ K/W, respectively. This means a reduction in total insulation due to defined body motion by 22.0%, 25.3% and 26.8%, respectively. Considering the *I*_Tr_ to *I*_T_ ratio (*I*_Tr_/*I*_T_) of the complete garment ensembles (SC and CP), and the values drawn in ISO 9920 (Equation 32 and Figure 4 of [10]), then these values were comparable and stayed somewhat above 0.7.

However, insulation in different body parts could change from +6% (Head in AL) to −49% (Left Hand in AL). In SC and CP, the changes were from 0% (Head) to −48% (Right Hand) and from −2% (Head) to −39% (Upper arms), respectively. The results, especially from AL, show clearly the effect of body parts’ swinging radius or being rigidly fixed in the walking manikin tests. The biggest change is for hands and feet followed by arms and legs, then torso zones and, finally, the head. Variation with clothing is modified by body area coverage, e.g., asymmetrical protection of hand and lower leg in SC (Figure 1b), and air permeability of the layers in CP (Figure 1c,d). Part of the difference could be also related to a variation in local air velocity at specific zones. Total thermal insulation of the textile skin (TS; complete coverage of the body, including hands feet and head) was 0.131 m^2^ K/W.

For technical measurements and various model evaluations, we need to consider what differences between the zones or changes do not match the reality. This may be built in the established correction equations, e.g., for walking. We may need to consider applying wind during testing in order to compensate for that. For example, walking at 3.5 km/h with a manikin may require 1 m/s wind to simulate the realistic influence of the motion. The same question may be raised to some extent for validation of the manikin results by humans walking on a treadmill.

The corrected total evaporative resistance of TS, SC and CP was 8.2, 20.9 and 81.0 m^2^ Pa/W (SC and CP include the skin and air layer resistance). Regional total evaporative resistance of TS shifted from 4.6 (right hand, thinner cotton glove was used) to 10.3 (right lower leg) m^2^ Pa/W. As mentioned above, some effect could be related to a variation in local air velocity around specific zones, but in this case, also to thickness of the skin and some overlap of separate layers (gloves at hands and socks on feet for skin simulation). Values for different parts of SC varied from 6.0 (right hand, almost nude, but with slight coverage of the end of the sleeve) to 65.6 (feet with boots) m^2^ Pa/W. Variation in CP was from 20.4 (head with some parts uncovered) to above 500 (belly, two tight layers above each other). There has been a discussion among manikin testers on how potential exclusion of the zones not covered by wet textile skin may affect the total evaporative resistance. Here, the total; total excluding the head, hands and feet; total excluding the hands and feet and total excluding the hands were calculated. The difference from the total was, on average, 2.0% (8.3−8.4 m^2^ Pa/W), 2.2% (20.6−22.2 m^2^ Pa/W) and 0.0% (73.7−92.2 m^2^ Pa/W) for TS, SC and CP, respectively, showing the influence of even or uneven evaporative resistance distribution; i.e., depending on tested clothing, the elimination of some body parts may influence increasing or decreasing the total insulation and thus, point to the importance of full-body coverage with wet skin.

In spite of these shortcomings, the current study managed to collect detailed data on real protective clothing used by workers in sugarcane fields. This study utilized only the values for the complete ensemble in a standard occupational heat strain model. However, the reported detailed data could be used in advanced human thermoregulatory models [19]. Additionally, detailed information on different body regions may allow improving the clothing for better ventilation and heat dissipation. This may be difficult in the case of chemical protection, while some new ideas may be generated for specific solutions, for example, using cooling systems in clothing [20,21,22].

### 4.2. Evaluation of Heat Strain with PHS

According to the model predictions, the heat exposure in chemical protective clothing was strongly limited by the increasing core temperature (Figure 3) and would be so under any (worse-case) high humidity scenario (Figure 5), too. Simultaneously, cane cutters’ core temperatures reached above 38 °C only at the highest activities and hottest periods of the hot day (Figure 3). In these cases, the continuous exposure should not exceed 50 min, and regular rest and drinking breaks are needed. The outcome clearly supports a known recommendation to have a long recovery/lunch breaks (>2 h) in well-ventilated areas in the shade and sufficient fluid replacements during the hottest period of the day. For the scenarios with high air humidity but lower solar loads, air temperatures above 34 °C may become a problem (Figure 5). In most of the evaluated conditions, dehydration can be a stronger limitation (Figure 4 and Figure 6): core temperature rises may trigger rest breaks, and during breaks, people drink. Alternatively, as the thirst sensation is not as strong a factor as core temperature rise, dehydration is harder to notice subconsciously [23]. The results from the current study strongly recommended that, depending on the weather conditions, more or less frequent drinking rest breaks should be enforced by the organization. As the PHS model calculates water loss, then advice for quantities and the frequency of drinking may be estimated. The PHS data also provides estimations of exposure time and rest break frequency based on core temperature calculations. This enables the evaluation of work situations, risk assessments and of the work/rest schedules. Lundgren et al. [15] has pointed out that the predictions for females have larger discrepancies from actual measurements; thus, the larger safety margins have to be applied for females and possibly for other specific population groups. Still, knowing the weather forecast or climate change predictions in addition to knowledge on clothing properties and workloads allows organizations to prepare for harsh conditions in advance and organize work practices that reduce negative health impacts with minimal losses in productivity. It is also important that risk assessments of work tasks are conducted regularly and in good time before any casualties occur, for example, according to the SOBANE strategy [24,25].

## 5. Conclusions

This study measured the properties of clothing used in sugarcane fields and utilized them in a standard tool for heat strain prediction. In spite of criticism on the PHS model, it allows a rough estimation of heat strain in the working population, as well as the preparation of countermeasures under hot conditions. This study also showed that weather data can effectively be utilized as input into the prediction models, and such automated inputs into a webtool or app can make a complex model into an easy-to-handle tool for practitioners, e.g., ClimApp [26]. The detailed clothing insulation and evaporative resistance values collected in the current study could be used in more advanced thermo-physiological models and, in combination with local weather data, could be used to support workplace policies and decision-making processes during hot weather or heatwaves. However, it must be considered that any model outcome must be utilized with care, as no model is perfect. Furthermore, the data used in model validations needs to be selected carefully, as test conditions may not always reflect real-world situations.

## Figures and Tables

**Table 1:** Input to predicted heat strain model (PHS) for the high humidity (RH = 70%) scenario.

Parameter	SC	CP
Acclimatization	1	1
Drink freely available	1	1
Body height	1.75	1.75
Body mass	70	70
Body surface area (calculated from mass and height, m^2^)	1.84	1.84
Posture: (1 = sitting, 2 = standing, 3 = crouching)	2	2
Air temperature	34 (28–36)	34 (28–36)
Air velocity	2.0	2.0
Metabolic energy production	250, 300, 350	200, 250, 300
Clothing basic insulation (1 clo = 0.155 m^2^ K/W)	0.69	1.14
Static moisture permeability index(calculated from insulation and evaporative resistance)	0.55	0.19
Fraction covered by reflective clothing	0	0
Angle between wind and walking direction	not used	not used
Walking speed	0.5	0.5
Mechanical power	0	0

Note: SC—sugarcane cutter and CP—chemical sprayer outfits, respectively.

**Table 2:** Total and total resultant insulation of the whole body, body regions and individual zones (m^2^ K/W) with percentual differences between static (*I*_T_) and dynamic (*I*_Tr_) values.

	Air Layer			Textile	Sugarcane Cutters				Chemical Sprayers			
Body Parts	*I*_T_ (=*I*_a_)	*I*_Tr_ (=*I*_ar_)	Diff (%)	skin*I*_T_	*I*_T_	*I*_Tr_	*I*_Tr_/*I*_T_	Diff (%)	*I*_T_	*I*_Tr_	*I*_Tr_/*I*_T_	Diff (%)
L.Hand	0.108	0.055	−48.7	0.135	0.180	0.114	0.63	−36.5	0.181	0.123	0.68	−32.1
Hands	0.100	0.053	−47.5	0.132	0.137	0.077	0.56	−44.0	0.172	0.115	0.67	−33.3
R.Hand	0.093	0.050	−46.5	0.129	0.109	0.057	0.52	−47.9	0.165	0.108	0.65	−34.8
Feet	0.103	0.057	−44.7	0.122	0.181	0.144	0.80	−20.0	0.208	0.149	0.72	−28.1
Lower Arms	0.101	0.056	−44.2	0.142	0.172	0.105	0.61	−39.3	0.259	0.165	0.64	−36.1
L.Lower leg	0.082	0.051	−38.3	0.111	0.168	0.128	0.76	−24.0	0.269	0.205	0.76	−23.8
Arms	0.104	0.069	−33.4	0.147	0.194	0.115	0.59	−40.5	0.258	0.160	0.62	−38.1
Upper arms	0.105	0.078	−25.8	0.149	0.209	0.122	0.59	−41.4	0.259	0.158	0.61	−39.3
Lower legs	0.082	0.063	−23.2	0.116	0.181	0.147	0.81	−18.9	0.255	0.212	0.83	−16.9
***Total***	***0.098***	***0.076***	***−22.0***	***0.131***	***0.191***	***0.143***	***0.75***	***−25.3***	***0.257***	***0.188***	***0.73***	***−26.8***
Legs	0.089	0.071	−19.8	0.118	0.206	0.154	0.74	−25.6	0.287	0.205	0.71	−28.6
Head, hands & feet excluded	0.093	0.076	−18.4	0.131	0.195	0.146	0.75	−25.3	0.278	0.200	0.72	−28.3
Belly	0.093	0.078	−16.2	0.121	0.331	0.261	0.79	−21.1	0.457	0.322	0.70	−29.6
Thighs	0.095	0.080	−15.2	0.121	0.229	0.160	0.70	−29.9	0.316	0.202	0.64	−35.9
R.Lower leg	0.082	0.075	−8.1	0.121	0.194	0.166	0.86	−14.4	0.241	0.219	0.91	−9.2
Buttocks	0.070	0.064	−7.9	0.107	0.253	0.220	0.87	−13.1	0.396	0.281	0.71	−29.1
Torso	0.092	0.086	−6.3	0.135	0.187	0.163	0.87	−12.8	0.283	0.226	0.80	−20.2
Back	0.093	0.090	−4.0	0.146	0.158	0.141	0.89	−11.3	0.228	0.190	0.83	−16.7
Chest	0.105	0.104	−0.6	0.152	0.160	0.141	0.88	−11.8	0.262	0.216	0.82	−17.7
Head	0.174	0.184	5.9	0.144	0.208	0.208	1.00	0.1	0.198	0.193	0.98	−2.2

Note: L—left, R.—right.

**Table 3:** Corrected total evaporative resistance of the whole body, body regions and individual zones (m^2^ Pa/W).

Body Parts	Textile Skin	Sugarcane Cutters	Chemical Sprayers
R.Hand	4.6	6.0	446.6
Hands	5.8	9.2	305.3
L.Lower leg	7.8	32.6	132.4
Legs	7.8	28.1	65.7
Thighs	7.4	27.7	56.7
L.Hand	7.7	16.1	245.8
Lower Arms	8.1	14.7	87.0
Belly	6.9	43.7	585.3
Lower legs	9.1	29.4	99.5
***Total***	***8.2***	***20.9***	***81.0***
Head, hands & feet excluded	8.3	21.2	92.2
Hands & feet excluded	8.4	20.6	73.7
Hands excluded	8.4	22.2	78.1
Feet	7.4	65.6	188.8
Arms	8.7	18.4	73.9
R.Lower leg	10.3	26.3	66.5
Torso	8.7	18.2	180.9
Back	9.0	13.8	165.1
Upper arms	9.1	21.2	70.4
Buttocks	9.2	32.3	98.6
Chest	9.2	14.6	203.1
Head	9.9	16.0	20.4

Note: L.—left, R.—right.
